# Real‐World Outcomes of Adjuvant Therapy in Stage III Melanoma and the Impact of Somatic Mutations

**DOI:** 10.1002/cam4.71410

**Published:** 2025-12-04

**Authors:** Derek Effiom, Tyler Aprati, Anastasios Karneris, Aleigha Lawless, Tatyana Sharova, Rebecca Johnson, Alexander Menzies, Georgina Long, Benjamin Park, Seungyeon Jung, Douglas Johnson, David Liu, Xue Bai, Keith Flaherty, Ryan Sullivan, Genevieve M. Boland, Sonia Cohen

**Affiliations:** ^1^ Division of Gastrointestinal and Oncologic Surgery, Department of Surgery Massachusetts General Hospital Boston Massachusetts USA; ^2^ Harvard Medical School Boston Massachusetts USA; ^3^ Department of Medical Oncology, Center for Immuno‐Oncology Dana‐Farber Cancer Institute Boston Massachusetts USA; ^4^ Broad Institute of MIT and Harvard Cambridge Massachusetts USA; ^5^ Melanoma Institute Australia The University of Sydney North Sydney New South Wales Australia; ^6^ Faculty of Medicine & Health The University of Sydney, Charles Perkins Center, Royal North Shore & Mater Hospital Sydney New South Wales Australia; ^7^ Department of Medicine Vanderbilt University Medical Center Nashville Tennessee USA; ^8^ Division of Medical Oncology, Department of Medicine Massachusetts General Hospital Boston Massachusetts USA

**Keywords:** anti‐PD1, BRAF, immunotherapy, melanoma, RFS, stage III

## Abstract

**Purpose:**

A significant proportion of patients with locoregional (stage III) cutaneous melanoma recur despite adjuvant systemic therapy. Staging criteria and surgical nodal management have changed since the trials were completed. Data assessing the effect of systemic therapy compared to surveillance are limited, and factors associated with recurrence are unclear. We assessed the efficacy of adjuvant systemic therapy in real‐world patients and assessed whether baseline genomic characteristics could prognosticate or predict benefit from therapy.

**Methods:**

We collected demographic, histopathologic, clinical, and genomic data for patients diagnosed with stage III cutaneous melanoma. Outcomes of interest were recurrence‐free survival (RFS) and distant‐metastasis‐free survival (DMFS). Survival analysis was performed using the Kaplan–Meier method with log‐rank analysis. Univariate and multivariate analyses were performed using a Cox regression analysis.

**Results:**

Two hundred and fifteen patients were included, of which 65 and 76 were treated with BRAF/MEK inhibitors (BRAFi/MEKi) and anti‐PD1 adjuvant systemic therapy respectively. Seventy four underwent active surveillance. Both adjuvant therapies reduced the hazard of recurrence when compared to patients undergoing active surveillance: anti‐PD1 HR: 0.32 (*p* < 0.01) and BRAFi/MEKi HR: 0.39 (*p* = 0.03). Anti‐PD1‐treated patients with a BRAF V600 mutation had a shorter RFS than patients with BRAF WT melanoma (*p* < 0.01); this was validated in external data where the presence of a BRAF V600 mutation was associated with an increased hazard recurrence (HR: 2.1, *p* = 0.025).

**Conclusion:**

Adjuvant systemic therapy improved RFS in our cohort. We found that BRAF V600 mutation was associated with a worse RFS for adjuvant anti‐PD1 monotherapy. The effect of BRAF mutation on the response to anti‐PD1 therefore may be considered when choosing between adjuvant anti‐PD1 and BRAFi/MEKi for patients with BRAF V600 mutant melanoma.

## 1 Introduction

Over 100,000 new cases of melanoma are estimated to be diagnosed annually, making it the 5th most common cancer in the United States. Although melanoma comprises only 1% of all skin cancers, it is responsible for 75% of all skin cancer‐related deaths [1]. Early‐stage melanoma (stages I and II) has a 5‐year overall survival (OS) rate of 98.4%. In contrast, survival rates for patients with locoregional metastasis (stage III) and distant metastasis (stage IV) are reported to be 63.6% and 22.5%, respectively, emphasizing the need for effective systemic therapies in advanced settings [2].

Systemic therapies available to patients with resected stage III melanoma in the adjuvant setting include immune checkpoint inhibitors (ICI) and BRAF/MEK inhibitors (BRAFi/MEKi). Previous trial data showed that adjuvant Ipilimumab (anti‐CTL4) improved recurrence‐free survival (RFS) outcomes compared to placebo [3]. Checkmate‐238 showed adjuvant Nivolumab (anti‐PD1) increased 4‐year RFS rates (51.7%) compared to Ipilimumab (41.2%) [4]. Additionally, Pembrolizumab (anti‐PD1) showed greater 7‐year RFS when compared to placebo [5]. Final analysis of adjuvant Dabrafenib and Trametinib (BRAFi/MEKi) at 10‐year follow‐up showed a significantly higher RFS compared to placebo (48% vs. 32%) and an OS benefit in patients with V600E mutations [6]. Notably, both COMBI‐AD and Keynote‐054 mandated completion lymphadenectomy (CLND) for patients with nodal metastases, which is no longer standard surgical practice. The landmark MSLT‐II trial showed that nodal basin observation instead of immediate CLND in patients with a positive sentinel lymph node biopsy (SLNB) resulted in no difference in melanoma‐specific survival and reduced surgical morbidity [7]. This resulted in widespread de‐escalation of surgery, with most patients now undergoing nodal surveillance rather than CLND for microscopic nodal disease [8, 9]. Moreover, stage IIIA melanoma patients with a nodal metastasis burden of less than 1.0 mm on sentinel lymph node biopsy were excluded from these adjuvant trials but adjuvant systemic therapy is approved in this setting under current management guidelines. These updates in management pose a clinical challenge for current decisions regarding the adjuvant treatment of patients with resected stage III melanoma [10].

This study aimed to analyze a real‐world cohort treated under current surgical and staging paradigms. The effect of adjuvant systemic therapy on patient outcomes and whether somatic mutation status at the time of diagnosis may be of predictive or prognostic relevance is evaluated.

## 2 Methods

### 2.1 Data Source & Study Design

A retrospective analysis was conducted on patients with stage III melanoma at a tertiary institution over 7 years (2015–2022). Medical chart review and data extraction occurred between November 2023 and April 2024. All patients were enrolled in an Institutional Review Board (IRB) protocol which allowed review of clinical data at Massachusetts General Hospital (MGH). Furthermore, all patients had prospective follow‐up.

The study employed the following inclusion criteria: (1) patients with stage III cutaneous or unknown primary melanoma as defined by the 8th edition of the American Joint Committee on Cancer (AJCC) [11], (2) patients treated with anti‐PD1, BRAFi/MEKi, or who underwent active surveillance; (3) patients with molecular sequencing to assess for somatic mutations at the time of stage III diagnosis. Exclusion criteria included lesions identified as acral lentiginous, mucosal, or uveal melanoma. Unclassified lesions in acral regions were treated as acral cutaneous melanomas. Furthermore, except for Interferon, prior systemic therapy was exclusionary.

### 2.2 External Validation

Clinical data from de‐identified patients diagnosed with stage III melanoma were provided by Melanoma Institute Australia (MIA, Australia) and Vanderbilt University Medical Center (VMC, USA). This cohort was used for validation purposes. All patients had local ethics approval in accordance with local regulations and guidance.

### 2.3 Outcomes

The primary outcome was RFS; the secondary outcome was distant‐metastasis‐free survival (DMFS). Recurrence was categorized as locoregional, distant or both. ‘Locoregional recurrence’ was defined as any regional soft tissue or nodal metastasis, ‘distant recurrence’ was defined as any distant soft tissue or visceral metastasis, with ‘both’ defined as locoregional and distant recurrences diagnosed simultaneously. Recurrence dates were based on the earliest suspicious lesion identified radiologically or clinically. RFS was the time from definitive surgery to first recurrence, and DMFS was calculated from the time of definitive surgery to the earliest detection of a distant recurrence. New primaries were not counted as recurrences.

### 2.4 Statistical Analysis

Descriptive statistics were used to summarize demographic, histopathological, clinical, and genomic variables. Fisher's exact test and Pearson's Chi‐squared test were used for univariate analysis of categorical variables. Survival outcomes were assessed using the Kaplan–Meier method and the Log‐rank test to investigate statistical differences. Prognostic variables were identified by univariate and multivariate Cox regression analyses. All *p* values were two‐sided and deemed statistically significant with *p*‐value(s) < 0.05. Data collection was performed using Microsoft Excel and analysis in R software system (packages: ggplot2, survminer, survival, and tidymodels).

## 3 Results

### 3.1 Baseline Characteristics

A total of 215 patients diagnosed with stage III cutaneous melanoma were included (Table S1): 74 (34.4%) were treated with surgery alone and received no adjuvant systemic therapy thereafter, 65 (30.2%) received adjuvant therapy with a BRAFi/MEKi, and 76 (35.3%) received adjuvant therapy with anti‐PD1 monotherapy. There were 116 (54.0%) males and 99 (46.0%) females; 94% of individuals identified as white. The median age at surgery was 61 (IQR: 47.5–69.0) years old. The BRAFi/MEKi cohort was younger with a median age of 54 years (IQR: 38–64, *p* < 0.001), and more likely to be female (*p* < 0.01). One hundred and ninety‐one (88.8%) patients were stage III at the time of initial melanoma diagnosis, with 24 (11.2%) being upstaged following recurrence. The primary tumor was cutaneous in 192 (89.3%) of patients with the remaining 23 (10.7%) diagnosed as tumors of unknown primary. Stage IIIC was the most common stage at 47.0%, followed by stage IIIB, IIIA and IIID at 31.2%, 18.6% and 3.2% respectively (Table 1). Furthermore, stage IIIA melanomas were less common in the anti‐PD1 cohort compared to other groups (*p* < 0.01). The median thickness was lower in the surveillance cohort at 2.3 mm (IQR: 1.4–3.1 mm), compared to 2.5 mm (IQR: 1.4–4.5 mm) and 2.9 mm (IQR: 1.8–4.6 mm) in the BRAFi/MEKi and anti‐PD1 cohorts respectively; the difference in tumor thickness between surveillance and anti‐PD1 treated patients was significant (*p* = 0.046). Ulcerated primary tumors were more frequent in the BRAFi/MEKi and anti‐PD1 groups, with rates of 35.4% and 39.5% compared with 20.3% in the surveillance group; similarly, the difference between anti‐PD1 and surveillance cohorts was significant (*p* = 0.035). No statistical difference was found between groups in tumor location, histological subtype, lymphovascular invasion, mitotic rate or tumor infiltrating lymphocytes (*p* > 0.5, Table S1). A BRAF V600 mutation was present in 9 (11.8%) patients within the anti‐PD1 cohort and 22 (29.7%) in the surveillance cohort. An NRAS mutation was present in 32 (43.2%) surveillance patients and 28 (36.8%) anti‐PD1 treated patients. For NF1 mutation frequencies were 20 (27.0%) and 23 (30.3%) respectively (Table 1).

**TABLE 1:** Demographic and clinical characteristics.

Characteristic	BRAFi/MEKi *N* = 65	anti‐PD1 *N* = 76	Active surveillance *N* = 74
Age—median (IQR)^1^	54 (38, 64)	61 (53, 68)	69 (56, 74)
Gender			
Female	42 (64.6)	32 (42.1)	25 (33.8)
Male	23 (35.4)	44 (57.9)	49 (66.2)
Stage III Presentation			
Initial	56 (86.2)	62 (81.6)	73 (98.6)
Recurrence	9 (13.8)	14 (18.4)	1 (1.4)
Nodal Disease			
Macroscopic	19 (29.2)	23 (30.3)	13 (17.6)
Microscopic	43 (66.2)	47 (61.8)	46 (62.2)
None	3 (4.6)	6 (7.9)	15 (20.3)
Surgery^2^			
SLNB	44 (67.7)	44 (57.9)	55 (74.3)
SLNB & CLND	2 (3.1)	3 (3.9)	2 (2.7)
TLND	16 (24.6)	25 (32.9)	11 (14.9)
Other	3 (4.6)	4 (5.3)	6 (8.1)
Stage^3^			
IIIA	14 (21.5)	1 (1.3)	25 (33.8)
IIIB	15 (23.1)	31 (40.8)	21 (28.4)
IIIC	33 (50.8)	41 (53.9)	27 (36.5)
IIID	3 (4.6)	3 (3.9)	1 (1.4)
Treatment			
Completed	31 (47.7)	34 (44.7)	Not Applicable
Discontinued due to progression	4 (6.2)	8 (10.5)	Not Applicable
Discontinued due to toxicity	22 (33.8)	24 (31.6)	Not Applicable
On‐going	1 (1.5)	1 (1.4)	Not Applicable
Other^4^	7 (10.8)	9 (11.8)	Not Applicable
Systemic therapy alteration			
Dose reduction – yes	38 (58.5)	0 (0.0)	0 (0.0)
Drug hold – yes	52 (80.0)	15 (19.7)	0 (0.0)
Not applicable	9 (13.8)	61 (80.3)	74 (100)
Steroid use for adverse event			
Yes	13 (20.0)	22 (28.9)	0 (0.0)
No	46 (70.8)	19 (25.0)	0 (0.0)
Not applicable	6 (9.2)	35 (46.1)	74 (100)
BRAF			
V600 mutant	65 (100)	9 (11.8)	22 (29.7)
Non‐V600 mutant	0 (0.0)	3 (3.9)	3 (4.1)
Wild type	0 (0.0)	64 (84.2)	49 (66.2)
NRAS			
Mutant	0 (0.0)	28 (36.8)	32 (43.2)
Wild type	65 (100)	48 (63.2)	42 (56.8)
NF1			
Mutant	4 (6.2)	23 (30.3)	20 (27.0)
Wild type	61 (93.8)	53 (69.7)	54 (73.0)

*Note:* Percentages may exceed 100% due to rounding. BRAF, NRAS and NF1 mutations refer to somatic mutations.

Abbreviations: CLND, completion lymphadenectomy; SLNB, sentinel lymph node biopsy; TLND, therapeutic lymphadenectomy.

^1^ Median age at time of surgery.

^2^ In addition to wide local excision (WLE) for patients with a known primary melanoma.

^3^ According to the 8th edition AJCC classification.

^4^ Other includes but is not limited to, patient decision, patient and physician agreement, poor patient condition and covid‐related concerns.

### 3.2 Treatment Outcomes

One hundred and fifty (69.8%) patients within this cohort underwent SLNB as part of their routine clinical care; this was followed by CLND in 7 (3.3%) patients. Upfront therapeutic lymphadenectomy (TLND) was performed in 52 (24.1%) patients due to clinically evident disease on presentation (Table 1). Lymphadenectomy, either upfront or completion, was higher in the anti‐PD1 cohort (36.8%) compared to the BRAFi/MEKi (27.7%) cohort with no statistically significant difference between groups. However, the comparison between anti‐PD1 (36.8%) and surveillance patients (17.6%) was statistically significant (*p* = 0.008). In the anti‐PD1 cohort, 49 (64.5%) patients received Pembrolizumab and 27 (35.5%) Nivolumab. Dabrafenib plus trametinib was prescribed for 64 (98.5%) of the 65 patients in the BRAFi/MEKi group, with the remaining patient receiving Encorafenib and Binimetinib. Two patients (2.7%) within the surveillance group received prior Interferon therapy in the adjuvant setting. The median treatment duration was 10.6 months (IQR: 3.1–12.3) for BRAFi/MEKi and 9.8 (IQR: 4.7–11.7) months for anti‐PD1 treated patients. Furthermore, adjuvant therapy was completed in 31 (47.7%) and 34 (44.7%) patients in the BRAFi/MEKi and anti‐PD1 cohorts respectively. Toxicity was the most common cause of treatment cessation with rates of 33.8% in the BRAFi/MEKi and 31.6% in the anti‐PD1 cohort. In patients who prematurely discontinued adjuvant therapy due to toxicity, the median treatment duration was 2.8 months (IQR: 1.8–3.4) and 4.7 months (IQR: 2.3–8.8), for patients treated with BRAFi/MEKi and anti‐PD1 respectively. Fifty‐two patients in the BRAFi/MEKi group and 15 patients in the anti‐PD1 group experienced drug holds during their treatment course. Specifically, within the BRAFi/MEKi group, 38 (58.5%) patients had dose reductions with or without drug holds. Steroid administration for adverse events occurred in 13 (20.0%) and 22 (28.9%) of the BRAFi/MEKi and anti‐PD1 treated patients (Table 1).

### 3.3 Patterns of Recurrence

**FIGURE 1:**
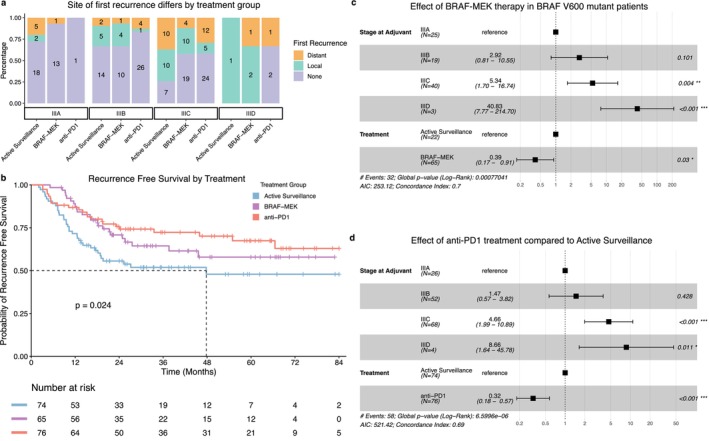
Treatment impacts recurrence‐free survival. (a) Bar chart of the proportions of recurrences and the site of first recurrence, grouped by stage and adjuvant therapy status. Patients with synchronous distant and local recurrences were noted as distant. The numbers within the bars represent the number of patients within that subgroup. (b) Kaplan Meier plot of recurrence‐free survival stratified by treatment group. The median RFS was not reached in patients receiving adjuvant treated patients; anti‐PD1 therapy significantly improved RFS compared with surveillance only. (c) Forest plot of cox proportional hazard model of recurrence free survival in BRAFi/MEKi treated patients compared with BRAF V600 mutant patients who underwent active surveillance only. BRAFi/MEKi adjuvant therapy reduces hazard of recurrence in BRAF V600 mutant melanoma patients compared with surveillance alone. (d) Forest plot of a Cox proportional hazard model of recurrence free survival in anti‐PD1 treated patients compared with patients who underwent active surveillance only. Anti‐PD1 treated patients had a significantly lower hazard of recurrence compared with patients managed with surveillance only.

The median follow‐up across the cohort was 37.8 months (IQR: 24.5–62.4), and 81 (37.7%) recurrences were observed. Twenty‐three (35.4%) patients in the BRAFi/MEKi experienced recurrence of their melanoma; 23 (30.3%) in the anti‐PD1 and 35 (47.3%) in the surveillance groups respectively. Of those who recurred, 46.9% did so within 1 year, with almost all recurring within 3 years (92.6%). Furthermore, the site of first recurrence was locoregional only in 16 (69.6%) of BRAFi/MEKi treated patients, with the inverse pattern observed in the anti‐PD1 group (*n* = 6, 26.1%); isolated distant recurrences were 21.7% and 60.9% respectively (Figure 1a). Similar rates were noted in the surveillance cohort, with 18 (51.4%) and 15 (42.9%) patients having the first recurrence as locoregional and distant respectively. Synchronous locoregional and distant recurrences were observed in 8.7%, 13.0% and 5.7% of BRAFi/MEKi, anti‐PD1 and active surveillance patients respectively. Overall survival following recurrence was not influenced by the site of the first recurrence, either at a cohort level or within individual groups (Figure S2).

### 3.4 Survival Outcomes

At 36 months, the RFS rate was 63.0% (95% CI: 57–70) and the DMFS was 78.0% (95% CI: 72–84) for the entire cohort. The median time to recurrence was 10.0 months (IQR: 7.0–15.2 months) in the active surveillance cohort, 14.0 months (IQR: 5.3–21.3 months) in the anti‐PD1 cohort and 16.1 months (IQR: 11.2–21.4 months) in the BRAFi/MEKi cohort; the median RFS was 47.8 months (IQR: 18.2—NR) in the surveillance group and was not reached in either adjuvant group (Figure 1b). Unadjusted analysis showed no statistical difference in RFS between the BRAFi/MEKi arm and patients with BRAF V600 mutation who underwent active surveillance; however, when controlling for stage, BRAFi/MEKi was associated with a significantly lower risk of recurrence (HR: 0.39, *p* = 0.03, Figure 1c). Anti‐PD1 therapy was shown to significantly reduce the risk of recurrence (HR: 0.32, *p* < 0.001) compared to active surveillance alone and prolong RFS (*p* = 0.012). Within the BRAFi/MEKi group, patients who prematurely discontinued therapy due to adverse events had a shorter RFS compared to those who completed (*p* < 0.01); however, similar analysis in the anti‐PD1 group displayed no statistically significant difference (*p* = 0.75, data not shown).

### 3.5 Survival Stratified by Mutation

**FIGURE 2:**
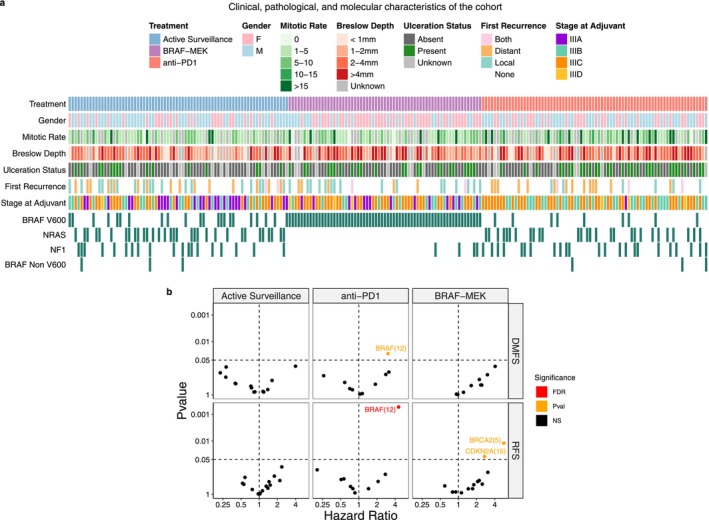
Baseline genomic status can influence survival outcomes. (a) CoMut plot of the entire study cohort (*n* = 215). Each column represents a patient, and each row represents a distinct variable. (b) Scatterplot of hazard ratios versus *p*‐value, categorized by survival outcome and treatment. Among patients treated with anti‐PD1, a BRAF mutation was associated with a significantly higher hazard. The orange color indicated significant results from univariate analyses, while red denotes results that remained significant after FDR correction. Abbreviations: FDR, False Discovery Rate; NS, non‐significant; pval, *p* value.

**FIGURE 3:**
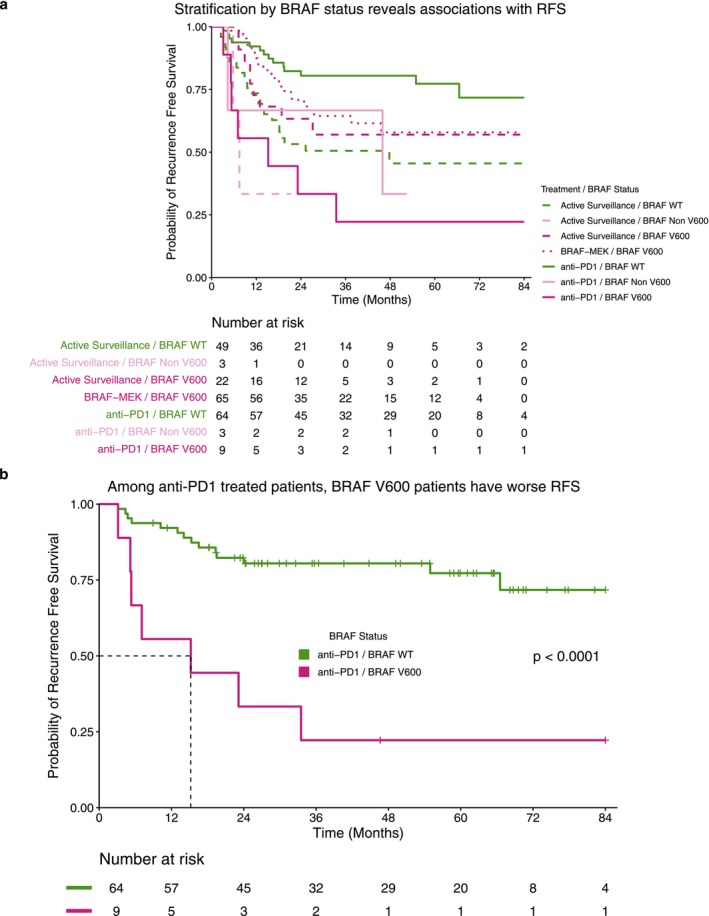
BRAF V600 mutations impact recurrence‐free survival in anti‐PD1 treated patients. (a) Recurrence‐free survival stratified by treatment and BRAF mutation status. (b) Recurrence‐free survival of stage III cutaneous melanoma patients treated with adjuvant anti‐PD1 therapy stratified by BRAF status. Within the anti‐PD1 cohort, patients with a BRAF V600 mutation demonstrated a significant reduction in recurrence‐free survival.

For each gene mutated in two or more patients, an unbiased univariate Cox regression analysis was performed comparing patients with a mutation to those with wild type (WT) melanomas (Table 2). This analysis was stratified by treatment group and outcomes of interest were RFS and DMFS (Figure 2). To account for multiple comparisons, a false discovery rate (FDR) correction was applied. These analyses identified mutations in BRAF (*n* = 12) as predictive of reduced benefit from anti‐PD1 in our stage III cohort (Figure 3a). Of those patients, 9 had a mutation in the V600 codon. As this mutation is targetable with BRAFi/MEKi and confers with therapeutic alternatives in the adjuvant setting, those 9 patients were the focus of subsequent analyses.

**TABLE 2:** Results of univariate Cox regression analyses.

Gene^*1*^	Treatment group^2^	Outcome tested	Hazard ratio (95% CI)	*p* (unadjusted)	*p* (adjusted)^*3*^
BRAF	anti‐PD1	RFS	4.6 (1.9–10.7)	< 0.001	0.008
BRAF	anti‐PD1	DMFS	3.0 (1.1–8.2)	0.028	0.426
BRCA2	BRAFi/MEKi	RFS	5.6 (1.5–21.9)	0.012	0.169
CDKN2A	BRAFi/MEKi	RFS	2.7 (1.9–10.7)	0.038	0.267

Abbreviations: CI, confidence interval; DMFS, distant‐metastasis‐free survival; RFS, recurrence‐free survival.

^1^ Only genes with statistically significant results following univariate analysis are shown. There were 12, 5 and 15 patients with a BRAF, BRCA2 and CDKN2A mutation respectively.

^2^ Patients with somatic mutations were compared with patients with wild‐type melanoma.

^3^ *p* values were adjusted using the false discovery rate correction.

In patients with BRAF V600 mutations, BRAFi/MEKi therapy resulted in a significantly longer RFS compared to anti‐PD1 treated patients (*p* = 0.0048). In patients treated with anti‐PD1 monotherapy (*n* = 76), those with BRAF V600 mutations (*n* = 9) had a significantly shorter RFS (*p* < 0.0001, Figure 3b) and an increased hazard of recurrence (HR 5.4, CI: 2.2–14.0, *p* < 0.001) compared to patients with BRAF WT melanomas treated with anti‐PD1 (*n* = 64). In addition, when accounting for age, the HR increased to 6.2 (CI: 2.3–16.5, *p* < 0.001, data not shown). Furthermore, no significant differences in assessed baseline characteristics were observed between groups (Table S1).

### 3.6 Validation of Results

**FIGURE 4:**
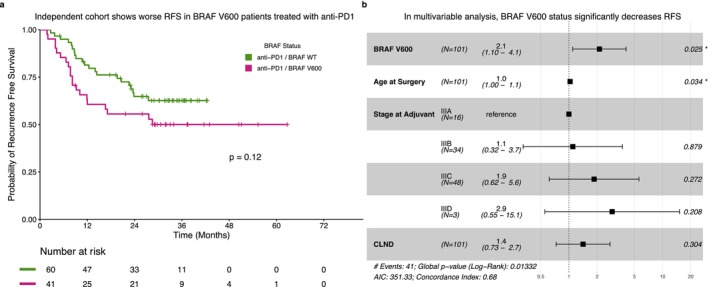
Patients with a BRAF V600 mutation display worse RFS in an independent stage III cohort. (a) Recurrence‐free survival of stage III melanoma patients (*N* = 101) stratified by BRAF mutation status. Despite separation of the Kaplan–Meier curves, log‐rank showed no statistical significance. (b) Forest plot of a multivariable Cox proportional hazard model of recurrence‐free survival. Multivariable analysis demonstrated patients with a BRAF V600 mutation have a significant increase in hazard of recurrence.

To validate our finding that BRAF mutation stratifies RFS in patients treated with adjuvant anti‐PD1, we reviewed an external cohort of patients with stage III cutaneous melanoma (*n* = 101, Table S2). The RFS rate at 36 months was 63% (CI: 51%–77%) in patients without a BRAF V600 mutation and 50% (CI: 37%–68%) in patients with. Log‐rank analysis comparing the survival curves between groups did not reach significance (*p* = 0.12, Figure 4a). However, patients with V600 mutations were significantly younger, with a median age of 56 (IQR: 39–61) years compared to 63 (IQR: 57–71) years in patients with WT melanomas (*p* = 0.002). Multivariable Cox regression analysis controlling for factors including age showed BRAF V600 mutation to be associated with an increased hazard for recurrence, consistent with our internal institutional data (HR: 2.1, *p* = 0.025, Figure 4b).

## 4 Discussion

This study reports real‐world outcomes of resected stage III cutaneous melanoma patients. Prospective trials that led to the approval of adjuvant systemic therapy for resected stage III melanoma used outdated staging criteria and mandated that all patients with a positive SLNB undergo CLND. Patient staging and surgical management have since changed, leading to questions regarding the applicability of these trial data to today's resected stage III melanoma patients. Our real‐world cohort demonstrated that adjuvant systemic therapy was associated with prolonged RFS. Furthermore, we observed that RFS for anti‐PD1 treated patients with a BRAF V600 mutation was less than the RFS of anti‐PD1 treated patients without a BRAF mutation. These observations are limited by the retrospective nature of the study. All patients in the observation cohort came from a single center; therefore practice patterns and year of diagnosis may bias results. We observed relative increases in the number of patients electing for active surveillance during the COVID pandemic years (Figure S3). In this reported cohort, TLND or CLND were performed in a total of 59 (27.4%) patients; therefore, as the nodal basins were not extensively sampled, this limits accurate pathologic staging. Whilst this reflects current clinical practice, it differs from the RCT investigating the efficacy of adjuvant systemic therapies. Finally, the number of patients with a BRAF mutation within the surveillance and anti‐PD1 cohorts was small, limiting the scope of analyses. We attempted to address these limitations with analyses of an external cohort.

As noted, we observed that adjuvant systemic therapy was associated with a RFS and DMFS benefit as previously reported in multiple studies. Keynote‐054 which compared Pembrolizumab against placebo in resected stage III melanoma reported 12‐ and 18‐month RFS rates of 75.4% and 71.4% in the Pembrolizumab arm [12]. Our findings were not dissimilar with 87% and 74% RFS at 12‐ and 24‐months. Similarly, COMBI‐AD investigated the effect of BRAFi/MEKi compared with placebo in stage III patients and showed RFS rates of 88% and 67% at 12‐ and 24‐months respectively [13]. We observed a RFS of 87% and 71% at 12‐ and 24‐months respectively in the BRAFi/MEKi group. Dima et al., conducted a retrospective review of 130 patients diagnosed with stage III melanoma treated with either anti‐PD1 (*n* = 100) or BRAFi/MEKi (*n* = 30) [14]. Patients treated with anti‐PD1 had 12 and 18‐month RFS rates of 79.3% and 70.2% respectively; this was 79.6% and 57.3% in BRAFi/MEKi treated patients. In a large multi‐institutional retrospective study of stage III melanoma patients, 12‐and 24‐month RFS rates were 63% and 50% for anti‐PD1 treated patients, and 91% and 67% for BRAFi/MEKi treated patients [15].

It is possible that the differences seen between adjuvant trial outcomes and retrospective studies are influenced by real‐world therapy adherence patterns. We reported higher than expected systemic therapy discontinuation rates of 33.8% and 31.6% in the BRAFi/MEKi and anti‐PD1 cohorts respectively. In Checkmate‐238 only 9.7% of patients treated with adjuvant Nivolumab prematurely discontinued due to toxicity, and the COMBI‐AD study reported that 26% of patients stopped combination therapy due to adverse events [4, 13]. Our results may reflect a lower threshold for cessation due to toxicity in everyday clinical practice for a preventative therapy. In line with this hypothesis, other retrospective studies have observed higher discontinuation rates than those reported in trials: in a population‐wide national database study of patients with resected stage III and IV cutaneous melanoma, 32% of patients discontinued adjuvant anti‐PD1 monotherapy due to toxicity [16]. Additionally, a recent Dutch registry study investigated outcomes of either anti‐PD1 or BRAFi/MEKi therapy in 646 patients with a BRAF mutated melanoma and stage III disease. Similar to our findings, 32.5% of BRAFi/MEKi treated patients discontinued therapy due to adverse events [17]. Despite higher rates of therapy discontinuation, adjuvant systemic therapy remained associated with a RFS benefit in our cohort of patients with stage III melanoma. No statistical difference was observed between patients who discontinued adjuvant anti‐PD1 and those who completed therapy, likely reflecting the ability of immunotherapy to elicit a durable response even with limited treatment duration.

A proportion of patients receiving adjuvant treatment would never have recurred following surgical management. Therefore, deciding who requires adjuvant therapy, with the potential for the risks of that treatment without certain benefit, is a unique challenge. Studies investigating predictive biomarkers for recurrence after adjuvant therapy are an area of active research [18]. Using molecular testing obtained as standard clinical care we performed an unbiased analysis of all reported somatic mutations in our cohort to investigate whether baseline pre‐treatment genomic features were associated with survival outcomes. Our analysis showed that patients with a BRAF V600 mutation treated with adjuvant anti‐PD1 monotherapy had a worse RFS than patients with BRAF WT melanoma. This difference was statistically significant despite the small number of patients with a V600 mutation. We repeated this specific analysis in a validation cohort of patients diagnosed with cutaneous stage III melanoma treated with adjuvant anti‐PD1; log‐rank analysis showed no statistical difference; however, regression analysis accounting for the significant difference in age between groups showed BRAF V600 mutation to be associated with an increased hazard for recurrence.

Previous adjuvant trials have not seen a similar outcome for patients with a BRAF V600 mutation treated with anti‐PD1 monotherapy. At five‐year follow‐up, a RFS of 54% was reported in patients with a BRAF V600 mutation treated with Pembrolizumab, similar to that of all Pembrolizumab‐treated patients (55%) [5]. Likewise, Checkmate‐238 and Checkmate‐915 show similar survival rates in patients treated with anti‐PD1 irrespective of BRAF status [4, 19]. However, randomization in all three phase III trials was designed to investigate the effect of treatment; it is unclear whether within anti‐PD1‐treated patients, BRAF V600 mutant and BRAF WT cohorts were comparable. In our validation analysis, accounting for other variables was required to isolate the effect of BRAF mutation.

Similarly, prior retrospective studies have not consistently addressed this question. Schumann et al. investigated the 12‐month RFS of 1198 stage III and IV cutaneous melanoma patients treated with adjuvant anti‐PD1 or Dabrafenib and Trametinib. In anti‐PD1 treated patients at 12‐month follow‐up patients who were BRAF WT had a significantly reduced hazard for recurrence compared to those harboring a BRAF mutation, in line with our findings [20]. Similarly, Dima et al. observed the median RFS to be 29.5 months for patients with a BRAF mutation treated with anti‐PD1, as compared to 64 months when all patients (those with and without a BRAF mutation) were analyzed as a single group [14]. Conversely, Rigo et al. in a similar cohort of patients with stage III and IV melanoma observed no difference in RFS between patients with a BRAF V600 mutation and patients with WT melanoma treated with either Nivolumab or Pembrolizumab. However, no multivariable analyses were performed to assess the specific effect of BRAF in this study [21]. In a similar analysis without multivariable adjustments, Holmstroem et al. also showed no statistical difference in RFS between patients with a BRAF mutation (*n* = 234) and patients without (*n* = 220) treated with anti‐PD1 [16]. None of these studies reported detailed data regarding the balance of other factors between the BRAF cohorts making definitive conclusions difficult.

The lack of clarity over whether BRAF status is associated with a reduced benefit from anti‐PD1 therapy in the stage III setting has clinical implications: patients with a V600 mutation are eligible for both anti‐PD1 and BRAFi/MEKi targeted therapy. The choice of adjuvant therapy is often driven by institutional practice patterns which may be influenced by trial data from other clinical settings [17]. The DREAMseq trial was a phase III trial that aimed to assess the best sequence of systemic therapy in a cohort of treatment‐naïve patients with a BRAF mutation diagnosed with unresectable melanoma [22]. Arm A received first‐line treatment with Ipilimumab and Nivolumab with Dabrafenib and Trametinib upon progression and Arm B was the reverse. Progression‐free survival (PFS) at 2 years was 41.9% and 19.2% in Arm A and Arm B respectively. Additionally, the duration of response was significantly greater in Arm A (median not reached), compared with patients in Arm B (median, 12.7 months). This showed a clear benefit of ICI as first‐line therapy for patients; however, combination Ipilimumab and Nivolumab was used and patients were unresectable; therefore the application of this to single‐agent anti‐PD1 in a resectable patient may not be appropriate.

Prospective phase III trials show worse PFS for patients with a BRAF mutation and advanced melanoma treated with anti‐PD1 monotherapy when compared to their BRAF WT counterparts. In the Checkmate 067 trial, patients with unresectable Stage III and IV melanoma with known BRAF status were randomized to either Ipilimumab plus Nivolumab, Nivolumab monotherapy or Ipilimumab monotherapy [23]. Interestingly, at 5‐year follow‐up PFS rates of 23% and 32% were reported in BRAF mutant patients and WT patients treated with Nivolumab respectively. However, when treated with combination Ipilimumab and Nivolumab, rates of 38% and 35% were reported. This suggests that BRAF V600 driven melanomas are associated with a reduced benefit from anti‐PD1 monotherapy in the advanced setting, whereas with the addition of Ipilimumab similar PFS rates are observed. A similar pattern has been observed in the neoadjuvant setting. The NADINA trial was a phase III trial which randomized enrolled patients with macroscopic stage III melanoma to either total lymphadenectomy (TLND) followed by adjuvant Nivolumab monotherapy or neoadjuvant Ipilimumab and Nivolumab with TLND; in the latter group adjuvant therapy was not prescribed if a major pathologic response was achieved following neoadjuvant therapy [24]. Adjuvant therapy with Nivolumab resulted in a 12‐month event‐free survival rate (EFS) of 52.2% and 62.4% in patients with a BRAF V600 mutation and patients with BRAF WT melanoma respectively. In the neoadjuvant group that received dual ICI, the 12‐month EFS was 83.5% in patients with a BRAF mutation which was comparable to the 83.9% noted in those without. This observation of BRAF status prognosticating for worse survival outcomes to anti‐PD1 monotherapy, albeit descriptive, appears to be consistent across multiple clinical contexts. In the stage III adjuvant setting the only approved use of adjuvant immunotherapy at this time is single‐agent anti‐PD1 monotherapy [10].

## 5 Conclusions

This study found that adjuvant therapy improved RFS and DMFS in a real‐world cohort of stage III cutaneous melanoma patients; this supports and is consistent with previously published adjuvant prospective trials. We observed that anti‐PD1 treated patients with a BRAF V600 mutation had a significantly shorter RFS than treated patients who were BRAF WT. Retrospective reviews have shown improved RFS for stage III patients with a BRAF V600 mutation when treated with BRAFi/MEKi compared with anti‐PD1 [15, 25]. Given the high frequency of BRAF mutation in cutaneous melanoma, greater clarity on the effect of BRAF mutation on adjuvant anti‐PD1 monotherapy will be of clinical utility.

## Author Contributions

**Derek Effiom:** conceptualization (equal), data curation (lead), formal analysis (equal), methodology (equal), visualization (lead), writing – original draft (lead), writing – review and editing (lead). **Tyler Aprati:** formal analysis (lead), methodology (lead), visualization (lead), writing – review and editing (supporting). **Anastasios Karneris:** data curation (supporting). **Aleigha Lawless:** data curation (supporting), project administration (lead). **Tatyana Sharova:** data curation (supporting), project administration (lead). **Rebecca Johnson:** data curation (supporting), validation (equal), writing – review and editing (supporting). **Alexander Menzies:** data curation (supporting), validation (equal), writing – review and editing (supporting). **Georgina Long:** data curation (equal), validation (equal), writing – review and editing (supporting). **Benjamin Park:** data curation (supporting), validation (equal). **Seungyeon Jung:** data curation (supporting), validation (equal). **Douglas Johnson:** data curation (supporting), validation (equal), writing – review and editing (supporting). **David Liu:** writing – review and editing (supporting). **Xue Bai:** data curation (supporting), validation (equal). **Keith Flaherty:** formal analysis (supporting), writing – review and editing (supporting). **Ryan Sullivan:** formal analysis (supporting), writing – review and editing (supporting). **Genevieve M. Boland:** conceptualization (lead), formal analysis (lead), methodology (lead), supervision (lead), writing – original draft (supporting), writing – review and editing (equal). **Sonia Cohen:** conceptualization (lead), formal analysis (lead), methodology (lead), supervision (lead), writing – original draft (supporting), writing – review and editing (lead).

## Funding

The authors have nothing to report.

## Ethics Statement

All patients within the observation cohort were consented to an Institutional Review Board (IRB) biobank protocol at Massachusetts General Hospital (MGH). For the validation cohort, local ethical approval was obtained in accordance with local regulation and guidance.

## Consent

The authors have nothing to report.

## Conflicts of Interest

D.J. Ad boards or consulting for AstraZeneca, BMS, Jackson Laboratories, Merck, Novartis, Pfizer, and Teiko, and research funding from BMS and Incyte. GVL is a consultant advisor for Agenus, Amgen, Array Biopharma, AstraZeneca, Bayer, BioNTech, Boehringer Ingelheim, Bristol Myers Squibb, Evaxion, GI Innovation, Hexal AG (Sandoz Company), Highlight Therapeutics S.L., Immunocore, Innovent Biologics USA, IOBiotech, Iovance Biotherapeutics, MSD, Novartis, PHMR Ltd., Pierre Fabre, Regeneron, Scancell, SkylineDX B.V. G.M.B. has sponsored research agreements through her institution with: Olink Proteomics, Teiko Bio, Intervenn Biosciences, Palleon Pharmaceuticals. She served on advisory boards for: Iovance, Merck, Moderna, Nektar Therapeutics, Novartis, and Ankyra Therapeutics. She consults for: Merck, InterVenn, Biosciences, Iovance and Ankyra Therapeutics. She holds equity in Ankyra Therapeutics. G.M.B. acknowledges support from the Adelson Medical Research Foundation (AMRF), the Patricia K. Donahoe Award from the Huiying Foundation, the Emma and Bill Roberts MGH Research Scholar Award, the Melanoma Research Alliance and the Department of Defense. S.C. acknowledges support from the Adelson Medical Research Foundation (AMRF) and the KL2 award from Harvard Catalyst/The Harvard Clinical and Translational Science Center (National Center for Advancing Translational Sciences, NIH Award KL2 TR002542).

## Supporting information


**Figure S1:** Patient inclusion flowchart.
**FIGURE S2:** Site of first recurrence did not impact overall survival.
**FIGURE S3:** Adjuvant therapy treatment patterns over time.

**Tables S1–S2:** cam471410‐sup‐0002‐Tables.docx.

## Data Availability

De‐identified data may be shared upon reasonable request to the corresponding author provided it is in accordance with regulatory requirements. The corresponding author does not have authority over data from other institutions; in such cases requests will need to be directed as appropriate.
