# Supplementary material for: Real‐World Outcomes of Adjuvant Therapy in Stage III Melanoma and the Impact of Somatic Mutations
Source: Cancer Med. 2025 Dec 4;14(23):e71410. doi: 10.1002/cam4.71410 (PMC12676251; doi:10.1002/cam4.71410)
Supplement: Supplementary file 1 — Figure S1: Patient inclusion flowchart. FIGURE S2: Site of first recurrence did not impact overall survival. FIGURE S3: Adjuvant therapy treatment patterns over time. [file CAM4-14-e71410-s002.pdf]

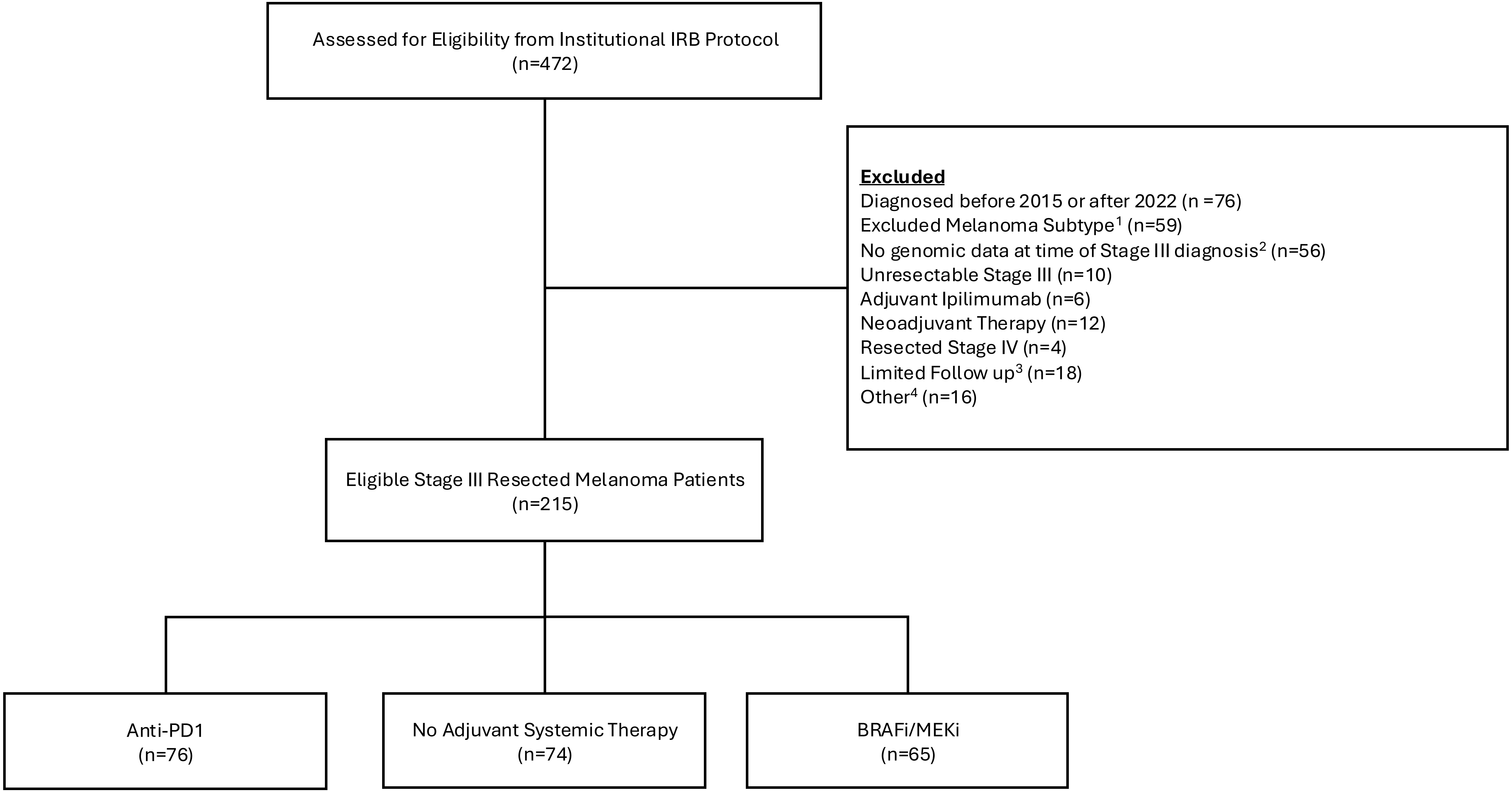
Assessed for Eligibility from Institutional IRB Protocol
(n=472)
Excluded
Diagnosed before 2015 or after 2022 (n =76)
Excluded Melanoma Subtype[1] (n=59)
No genomic data at time of Stage III diagnosis[2] (n=56)
Unresectable Stage III (n=10)
Adjuvant Ipilimumab (n=6)
Neoadjuvant Therapy (n=12)
Resected Stage IV (n=4)
Limited Follow up[3] (n=18)
Other[4] (n=16)
Eligible Stage III Resected Melanoma Patients
(n=215)
Anti-PD1
(n=76)
No Adjuvant Systemic Therapy
(n=74)
BRAFi/MEKi
(n=65)

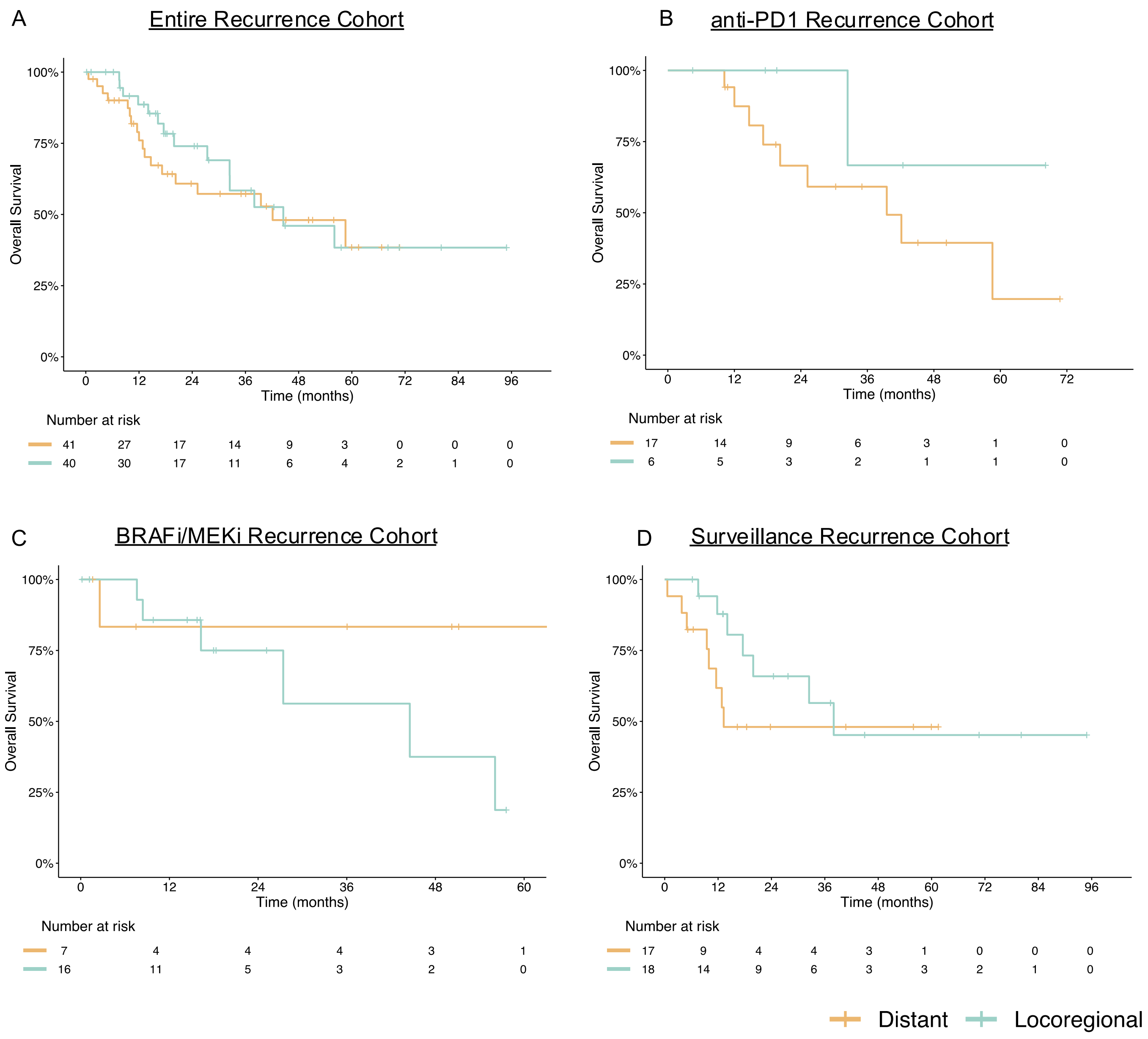
A
Entire Recurrence Cohort
Overall Survival
Time (months)
Number at risk
41 27 17 14 9 3 0 0 0
40 30 17 11 6 4 2 1 0
B
anti-PD1 Recurrence Cohort
Overall Survival
Time (months)
Number at risk
17 14 9 6 3 1 0
6 5 3 2 1 1 0
C
BRAFi/MEKi Recurrence Cohort
Overall Survival
Time (months)
Number at risk
7 4 4 4 3 1
16 11 5 3 2 0
D
Surveillance Recurrence Cohort
Overall Survival
Time (months)
Number at risk
17 9 4 4 3 1 0 0 0
18 14 9 6 3 3 2 1 0
Distant
Locoregional

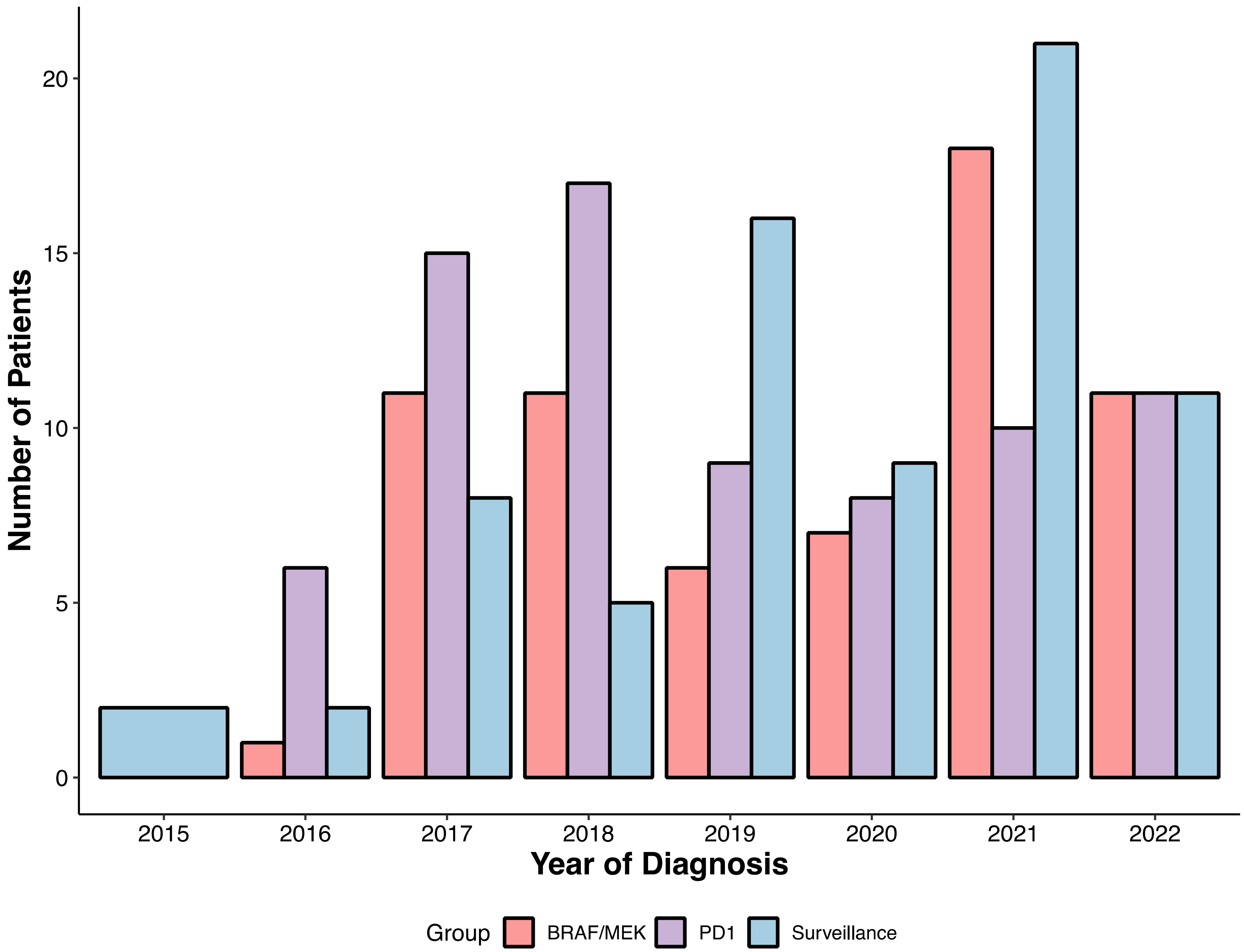
Number of Patients
0
5
10
15
20
2015
2016
2017
2018
2019
2020
2021
2022
Year of Diagnosis
Group
BRAF/MEK
PD1
Surveillance
