# Supplementary material for: Real‐World Outcomes of Adjuvant Therapy in Stage III Melanoma and the Impact of Somatic Mutations
Source: Cancer Med. 2025 Dec 4;14(23):e71410. doi: 10.1002/cam4.71410 (PMC12676251; doi:10.1002/cam4.71410)
Supplement: Supplementary file 2 — Tables S1–S2: cam471410‐sup‐0002‐Tables.docx. [file CAM4-14-e71410-s001.docx]

| **Supplemental Table 1** | **Entire Study cohort** | | | **anti-PD1 cohort** | |  |
| --- | --- | --- | --- | --- | --- | --- |
| **Characteristic** | **BRAFi/MEKi**  N = 65^1^ | **anti-PD1**  N = 76^1^ | **Active Surveillance**  N = 74^1^ | **BRAF WT**  N = 64^1^ | **BRAF V600 Mu**  N = 9^1^ | **BRAF Non-V600 Mu**  N = 3^1^ |
| **Race** |  |  |  |  |  |  |
| White | 60 (92.3) | 72 (94.7) | 70 (94.6) | 62 (96.9) | 7 (77.8) | 3 (100.0) |
| Non-White | 2 (3.1) | 1 (1.4) | 0 (0.0) | 1 (1.6) | 0 (0) | 0 (0) |
| Unknown | 3 (4.6) | 3 (3.9) | 4 (5.4) | 1 (1.6) | 2 (22.2) | 0 (0) |
| **Primary Melanoma Present** |  |  |  |  |  |  |
| Yes | 59 (90.8) | 69 (90.8) | 64 (86.5) | 58 (90.6) | 9 (100.0) | 2 (66.7) |
| No | 6 (9.2) | 7 (9.2) | 10 (13.5) | 6 (9.4) | 0 (0.0) | 1 (33.3) |
| **Primary Site** |  |  |  |  |  |  |
| Head and Neck | 10 (15.4) | 13 (17.1) | 12 (16.2) | 12 (18.8) | 0 (0.0) | 1 (33.3) |
| Upper Extremity | 5 (7.7) | 17 (22.4) | 12 (16.2) | 15 (23.4) | 2 (22.2) | 0 (0.0) |
| Trunk | 21 (32.3) | 21 (27.6) | 20 (27.0) | 16 (25.0) | 4 (44.4) | 1 (33.3) |
| Lower Extremity | 23 (35.4) | 16 (21.1) | 20 (27.0) | 13 (20.3) | 3 (33.3) | 0 (0.0) |
| Other | 0 (0.0) | 2 (2.6) | 0 (0.0) | 2 (3.1) | 0 (0.0) | 0 (00) |
| Unknown Primary | 6 (9.2) | 7 (9.2) | 10 (13.5) | 6 (9.4) | 0 (0.0) | 1 33.3) |
| **Histological Subtype** |  |  |  |  |  |  |
| SSM | 33 (50.8) | 34 (44.7) | 29 (39.2) | 28 (43.8) | 6 (66.7) | 0 (0.0) |
| NM | 11 (16.9) | 18 (23.7) | 13 (17.6) | 14 (21.9) | 2 (22.2) | 2 (66.7) |
| Lentigo | 1 (1.5) | 3 (3.9) | 3 (4.1) | 3 (4.7) | 0 (0.0) | 0 (0.0) |
| Desmoplastic | 0 (0) | 2 (2.6) | 0 (0.0) | 2 (3.1) | 0 (0.0) | 0 (0.0) |
| Nevoid | 1 (1.5) | 1 (1.3) | 0 (0.0) | 1 (1.6) | 0 (0.0) | 0 (0.0) |
| Spitzoid | 1 (1.5) | 0 (0.0) | 1 (1.4) | 0 (0.0) | 0 (0.0) | 0 (0.0) |
| Unclassified | 11 (16.9) | 9 (11.8) | 18 (24.3) | 8 (12.5) | 1 (11.1) | 0 (0.0) |
| Unknown | 7 (10.8) | 9 (11.8) | 10 (13.5) | 8 (12.5) | 0 (0.0) | 1 (33.3) |
| **Breslow Depth, mm - median (IQR)** | 2.5 (1.4 - 4.5) | 2.9 (1.8 - 4.6) | 2.3 (1.4 -3.1) | 2.8 (1.8 -4.3) | 2.5 (1.1 -5.5) | 4.1 (3.7 -4.6) |
| **Ulceration** |  |  |  |  |  |  |
| Present | 23 (35.4) | 30 (39.5) | 15 (20.3) | 26 (40.6) | 3 (33.3) | 1 (33.3) |
| Absent | 35 (53.8) | 37 (48.7) | 46 (62.2) | 31 (48.4) | 5 (55.6) | 1 (33.3) |
| Indeterminate/Unknown | 7 (10.8) | 9 (11.8) | 13 (17.6) | 7 (10.9) | 1 (11.1) | 1 (33.3) |
| **Mitotic Rate (per mm2), median (IQR)** ^2^ | 4.5 (2.0 -7.4) | 7.0 (3.0 - 14.0) | 5.0 (3.0 - 9.0) | 7.0 (4.0 -9.1) | 6.0 (3.0 -12.86) | 2.5 (2.25 - 2.75) |
| **Lymphovascular Invasion** |  |  |  |  |  |  |
| Absent | 46 (70.8) | 51 (67.1) | 53 (71.6) | 46 (71.9) | 5 (55.6) | 0 (0.0) |
| Present | 10 (15.4) | 14 (18.4) | 11 (14.9) | 11 (17.2) | 1 (11.1) | 2 (66.7) |
| Unknown | 9 (13.8) | 11 (14.5) | 10 (13.5) | 7 (10.9) | 3 (33.3) | 1 (33.3) |
| **T Cell Infiltration** |  |  |  |  |  |  |
| Present, brisk | 4 (6.2) | 6 (7.9) | 1 (1.4) | 6 (9.4) | 0 (0.0) | 0 (0.0) |
| Present, nonbrisk | 47 (72.3) | 46 (60.5) | 49 (66.2) | 39 (60.9) | 6 (66.7) | 1 (33.3) |
| Absent | 6 (9.2) | 12 (15.8) | 14 (18.9) | 9 (14.1) | 2 (22.2) | 1 (33.3) |
| Unknown | 8 (12.3) | 12 (15.8) | 10 (13.5) | 10 (15.6) | 1 (11.1) | 1 (33.3) |
| ^1^ n (%)  *^2^* Number of patients with documented mitotic rate included, 58, 66 and 64 patients for BRAFi/MEKi, anti-PD1 and active surveillance cohort, respectively.  Percentages may not sum to 100% due to rounding.  *Abbreviations: Mu mutant; NM, Nodular Melanoma; SSM, Superficial Spreading Melanoma; WT, wild type.* | | | | | | |

| **Supplemental Table 2: Demographic & clinical features of patients within the validation cohort.** | | | |
| --- | --- | --- | --- |
| Characteristic | V600 Mutation  N = 41 | Wild Type  N = 60 | p-value |
| Age – median (IQR) | 56 (39, 61) | 63 (57, 71) | 0.002 |
| Sex |  |  | 0.4 |
| Female | 10 (24%) | 19 (32%) |  |
| Male | 31 (76%) | 41 (68%) |  |
| Race |  |  | 0.4 |
| White | 40 (98%) | 60 (100%) |  |
| Non-White | 1 (2.4%) | 0 (0%) |  |
| Breslow Depth, mm – median (IQR)^1^ | 3.0 (1.7 – 4.6) | 3.0 (1.6 -5.0) | 0.9 |
| Ulceration |  |  | 0.7^2^ |
| Present | 18 (44%) | 19 (31%) |  |
| Absent | 21 (51%) | 27 (45%) |  |
| Unknown Primary / Unavailable^2^ | 2 (5%) | 14 (23%) |  |
| Stage |  |  | 0.7 |
| IIIA | 7 (17%) | 9 (15%) |  |
| IIIB | 12 (29%) | 22 (37%) |  |
| IIIC | 20 (49%) | 28 (47%) |  |
| IIID | 2 (4.9%) | 1 (1.7%) |  |
| Completion Lymphadenectomy |  |  | 0.9 |
| Yes | 17 (41%) | 26 (43%) |  |
| No | 24 (59%) | 34 (57%) |  |
| Adjuvant Radiotherapy |  |  | 0.079 |
| Yes | 6 (15%) | 3 (5.0%) |  |
| No | 34 (83%) | 57 (95%) |  |
| Unknown | 1 (2.4%) | 0 (0%) |  |
| Recurrence |  |  | 0.2 |
| Yes | 20 (49%) | 21 (35%) |  |
| No | 21 (51%) | 39 (65%) |  |
| *^1^ Patients with missing data (n=5) or unknown primary (n=9) were removed from this calculation.*  *^2^ Comparison included patients with known values only.*  All patients were diagnosed with stage III cutaneous melanoma and received adjuvant anti-PD1. | | | |
